# Ionizing radiation-induced cancer: perplexities of the bystander effect

**DOI:** 10.3332/ecancer.2023.1579

**Published:** 2023-07-20

**Authors:** Lakshmi Gopinathan, C Gopinathan

**Affiliations:** 1 Independent consultant, Navi Mumbai 400703, India; 2 Ex-Head, Chemistry Division, Bhabha Atomic Research Centre, Trombay, Mumbai 400085, India

**Keywords:** cancer, ionizing radiation, bystander effect, non-targeted effect

## Abstract

Ionizing radiation (IR) is a carcinogen. This has been established beyond doubt from many years of studies such as those conducted among the survivors of the atomic bomb attacks on Hiroshima and Nagasaki and later from the Chernobyl accident. Despite immense progress in the field of carcinogenesis, complete understanding of the underlying mechanisms behind IR-induced cancer remains elusive. In particular, the long gestation period between exposure to IR and the onset of cancer, frequently unpredictable, and sometimes lasting for many years, remains poorly understood. The centrality of DNA damage and misrepair in carcinogenesis research has not entirely benefited IR-induced cancer research and the past decade has seen a shift in understanding radiation-driven cellular mechanisms beyond simplistic models of targeted DNA damage. This paper presents a viewpoint on the gaps in our knowledge of IR-induced cancer with a focus on the non-targeted bystander effect, the mechanisms underlying which may be key to radiotherapeutic advances.

## Introduction

**Figure 1.**
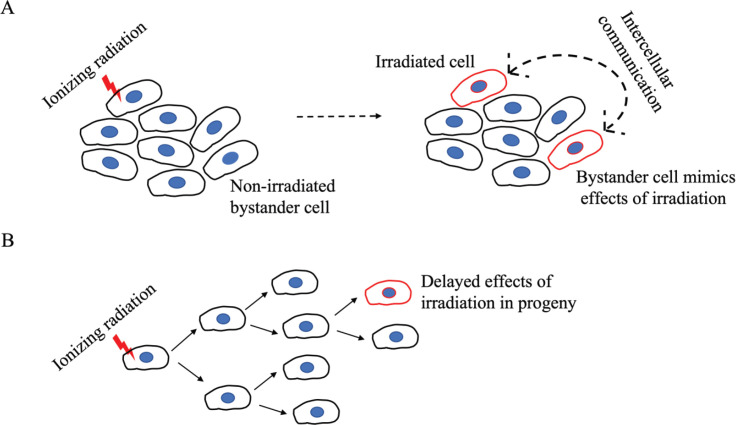
A schematic representation of the non-targeted effects of IR on bystander cells. (a): An irradiated cell communicates stress stimuli to a neighbouring non-irradiated cell which then mimics the outcome of IR. The bidirectional arrow indicates that the communication can be reciprocal such as the adaptive response seen in radiotherapy where bystander cells can diminish the damage response in irradiated cells. (b): The effects of IR are seen only in distant progeny.

In a nuclear weapon, several switches have to be activated in the right sequence for the bomb to explode. Carcinogenesis is similarly a multi-stage process where information from various damage centres has to be recalled in the right sequence for the cell to transition into a cancerous mode. Once the various damage sites have been created, the system is like a ticking time bomb waiting to explode. The series of sequential events such as tumour initiation, promotion and progression involving activation of oncogenes and inactivation of tumour-suppressing genes are well studied and have been extensively reviewed [1, 2]. The long latent period between exposure to ionizing radiation (IR) and the onset of cancer, which in the case of humans could run into decades, could be attributed to the duration of this sequence. However, the mutations and growth characteristics of tumours induced by radiation appear indistinguishable from spontaneous or other carcinogen-induced tumours that have quicker onset or similar multi-stage progression [3]. Further, the delayed occurrence of genetic aberrations in the progeny of irradiated cells and the occurrence of DNA damage in non-irradiated cells near the site of irradiation (Figure 1) strongly challenge a direct causal connectivity from radiation to DNA damage to cancer. In this scoping review, we discuss a few established as well as evolving insights that support a multifarious model of radiation carcinogenesis. We are hopeful that our perspective on IR-induced non-targeted effects can help researchers identify poorly understood aspects in the field and incite further research on the mechanisms of IR-induced cancer with potential implications in the radiotherapeutic management of cancer.

## Methodology

We searched PubMed using the terms ‘IR-induced cancer’, ‘IR-induced non-targeted effects’, ‘IR-induced non-targeted bystander effect’, and ‘IR carcinogenesis’ with no time limit to identify relevant studies. Taking into consideration our aim to provide a focused review, we have cited as many studies as possible, both original as well as review articles that offer a historical perspective of knowledge evolution as well as current understanding.

### IR-induced non-targeted effects

Mechanistic radiobiology studies for a long time focused on IR-induced DNA damage and misrepair as initiators of cancer. However, given the protracted gestation period for cancer development following IR exposure, radiation-induced initiating events are unlikely to be effects on specific genes that typify-targeted cancer onset. Rather, a delayed or an enduring event(s) with eventual tumorigenic implications is a more likely culprit. To this end, persistent genomic instability induced by radiation has gained acceptance over the last decade as the grounds for mutagenic events that are tumorigenic [4]. Mutagenic damage as such an indirect effect of radiation exposure is supported by experimental studies demonstrating the normal division of irradiated cells over several generations, with mutagenic change appearing only in distant progeny [5]. This presents a further challenge to the assumption of cancer initiation by radiation-induced direct DNA damage and is a curious phenomenon termed the bystander effect. In contrast to its use in psychology where the term refers to an apathetic state, the bystander effect in radiation biology refers to the active involvement of ‘bystander’ non-irradiated cells, located near the site of irradiation, in the development of cancer. Revealing an intricate web of cell-cell communication, irradiated cells convey stress stimuli to non-irradiated cells which then mimic the outcomes of radiation exposure in a delayed manner, sometimes only after several generations (Figure 1). Initially identified as chromosomal aberrations in the progeny of cells irradiated with a-particles [6, 7], the radiation-induced bystander effect (RIBE) or radiation-induced non-targeted effect, is now known to involve the gamut of IR-induced modifications such as mutations, chromosomal instability, apoptosis, epigenetic changes and altered cell signalling, thereby associating RIBE closely with several hallmarks of cancer [8, 9]. In contrast to the direct effects of radiation, RIBE exhibits a non-linear dose-response with effects seen at very low doses and may be linked to secondary cancers in patients who have undergone radiation treatment. These systemic long-range effects of irradiation, occurring at sites distant from the irradiated volume within the same organism are referred to as abscopal effects, and have led to the identification of inhibitors of RIBE to minimise irradiation risks for patients [10–12].

**Figure 2.**
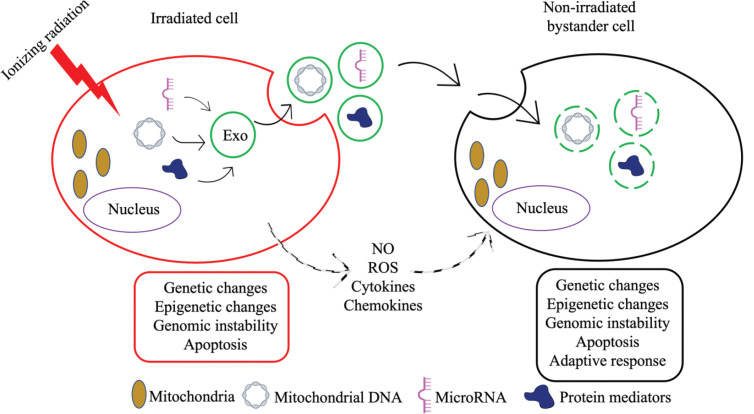
A schematic representation of the mechanisms of intercellular communication between an irradiated cell and a bystander cell. An irradiated cell can communicate stresses to a bystander cell via NO-and ROS-based signalling pathways, by the release of diffusible factors such as cytokines and chemokines, or by exosome (Exo)-mediated transport of cargo such as mtDNA, miRNA and protein mediators. In addition to mimicking IR-induced changes such as genetic changes, epigenetic changes and genomic instability, bystander cells may also exhibit adaptive responses such as those seen in response to radiotherapy.

Despite extensive research, RIBE remains a perplexing phenomenon and the underlying molecular pathways are not fully understood. Key mechanisms involve communication via intercellular gap junctions [13–15] or directly by the release of diffusible factors by irradiated cells [16, 17, reviewed in 18] (Figure 2). Gap-junction-mediated signaling can occur via ions such as calcium or small molecules such as nitric oxide (NO), while secretions into the extracellular compartment include cytokines such as transforming growth factor-β chemokines and reactive oxygen species (ROS) [19–22]. In addition, exosomes, a form of extracellular vesicles, have been proposed as transmitters of RIBE [23]. Early studies reported increased levels of Fas ligand, also called the ‘death ligand’, on extracellular vesicles in response to IR [24]. Since then, many types of exosome cargo such as mitochondrial DNA (mtDNA), non-coding RNAs, immune players, lipids and protein mediators have been implicated in RIBE (Figure 2) [25]. The persistent longevity of exosome-mediated RIBE was well demonstrated when non-irradiated cells treated with exosomes derived from the progeny of bystander cells also exhibited RIBE [26].

A demonstration of a role for mtDNA in RIBE came from experiments where exosomes or conditioned medium derived from irradiated cells depleted of mtDNA were unable to induce DNA damage in non-irradiated cells [27, 28]. In comparison to its nuclear counterpart which is protected by histones, mtDNA has been reported to be more sensitive to certain oxidative damages due to its proximity to the electron transport chain [29, 30]. The criticality of mitochondria in energy production and cellular metabolism suggests that exosome-mediated communication of mitochondrial dysfunction using mtDNA could represent an important facet of RIBE. In more recently identified mechanisms, biophotons, which are photons of light in the ultraviolet and low visible light range, have been proposed as intercellular communicators of radiation-induced stress and have been shown to incite bystander cells to release exosomes and disrupt mitochondrial oxidative phosphorylation [31, 32].

One of the most notable advances in understanding the role of non-coding RNAs in IR-induced cancer has been their detection in exosomes from irradiated cells [33]. Among the various classes of non-coding RNA, long non-coding RNA (lncRNA), microRNA (miRNA) and circular RNA have been studied the most in IR-induced carcinogenesis [34, 35]. Because of distinct changes in their expression upon radiation exposure, and due to their stability and abundance in serum and other biofluids, several of these non-coding RNAs have been proposed as diagnostic and prognostic biomarkers of radiation damage. While most research has focused on their utility in assessing response to radiotherapy, their roles in IR-induced cancer are less clear ([36–38] for lncRNA; [39–42] for miRNA; and [43–45] for circular RNA). Non-coding RNAs have been shown to respond to IR by modulating key pathways related to apoptosis, the cell cycle, DNA damage repair, glycolysis and autophagy [46–49]. One of the most abundant and conserved miRNAs regarded as an important cancer biomarker, miR-21, is a well-studied example whose increased expression in bystander cells is likely mediated through endocytic uptake by exosomes [50].

### Implications of radiation-induced non-targeted effects in cancer therapy

In addition to the studies that have focused on the damaging features of RIBE, some have also described alternate aspects such as stress resistance, adaptive responses to radiotherapy, increased cell proliferation and enhanced cell differentiation [5, 51, 52]. In this regard, it is important to note that the communication between irradiated and bystander cells can be reciprocal, whereby bystander cells can diminish the damage response in irradiated cells (Figure 1a) [10, 53]. Although studies on RIBE have yet to clearly define a mechanism, studying RIBE in relation to radiotherapy has revealed its immunomodulatory nature. Apart from cytokine or exosome-mediated immune modulation, T-cell activation via the presentation of tumour-associated antigens from IR-damaged cancer cells is an important consideration that is a likely feature of abscopal effects. The recruitment of leucocytes, maturation of antigen-presenting cells and activity of cytotoxic lymphocytes are key immune aspects shown to be modulated by radiotherapy [10]. The integration of this knowledge with cancer immunotherapy may prove valuable for therapeutic advances by assessing radiation risk and improving patient responses to radiotherapy. Greater understanding of immunomodulatory abscopal effects has led to studies investigating the combinatorial efficacy of cancer immunotherapy and radiotherapy in an effort to enhance abscopal antitumor effects, which are normally rare to occur [54, 55]. These include studies on the concurrent use of IR with checkpoint inhibitors, cytokines and cytotoxic immune therapy.

## Conclusion

We have strived to provide a critical overview that can clarify concepts and identify gaps in the current understanding of the mechanisms underlying IR-induced carcinogenesis. While evidence has accumulated for the criticality of non-targeted effects of IR, a comprehensive understanding of the molecular events leading to IR-induced cancer is lacking. How radiation-induced non-targeted effects influence the immune cell machinery and manifest as abscopal effects is also poorly understood. A deeper understanding of these concepts may prove instrumental in advancing the immunotherapeutic and radiotherapeutic management of cancer.

## Conflicts of interest

The authors do not have any conflict of interest to declare.

## Funding

The authors do not have any financial declaration.
